# Coronary flow velocity reserve and inflammatory markers in living kidney donors

**DOI:** 10.1016/j.ijcard.2020.08.013

**Published:** 2020-12-01

**Authors:** Ashwin Radhakrishnan, Anna M. Price, Luke C. Pickup, Jonathan P. Law, Kirsty C. McGee, Larissa Fabritz, Roxy Senior, Richard P. Steeds, Charles J. Ferro, Jonathan N. Townend

**Affiliations:** a Birmingham Cardio-Renal Group, Institute of Cardiovascular Sciences, University of Birmingham, Birmingham, United Kingdom; b Department of Cardiology, Queen Elizabeth Hospital, Birmingham, United Kingdom; c Department of Nephrology, Queen Elizabeth Hospital, Birmingham, United Kingdom; d Institute of Inflammation and Ageing, University of Birmingham, Birmingham, United Kingdom; e Institute of Cardiovascular Sciences, University of Birmingham, Birmingham, United Kingdom; f Cardiac Research Unit, Northwick Park Hospital, London, United Kingdom; g Department of Cardiology, Royal Brompton Hospital, London, United Kingdom

**Keywords:** Living kidney donors, Coronary microvascular dysfunction, Coronary flow velocity reserve, Inflammation, BP, Blood pressure, CAD, Coronary artery disease, CFR, Coronary flow reserve, CFVR, Coronary flow velocity reserve, CFV, Coronary flow velocity, CKD, Chronic kidney disease, CMD, Coronary microvascular dysfunction, CRIB-Donor, Chronic Renal Impairment in Birmingham - Donor study, CRIB-FLOW, Chronic Renal Impairment in Birmingham Coronary FLOW Reserve study, CRP, C reactive peptide, ECG, Electrocardiogram, eGFR, Estimated glomerular filtration rate, IL-1ra, Interleukin-1 receptor antagonist, IL-6, Interleukin-6, IL-8, Interleukin-8, hsCRP, High sensitivity C reactive peptide, LKD, Living kidney donors, LVH, Left ventricular hypertrophy, MCE, Myocardial contrast echocardiogram, MI, Mechanical index, SD, Standard deviation, SNS, Sympathetic nervous system, TNFα, Tumour necrosis factor α, TTE, Transthoracic echocardiogram, QEHB, Queen Elizabeth Hospital Birmingham, UK, United Kingdom

## Abstract

**Background:**

Coronary microvascular dysfunction is prevalent in chronic kidney disease (CKD), and may contribute to the development of myocardial dysfunction in CKD. Coronary flow velocity reserve (CFVR) is a marker of coronary microvascular function and falls with increasing CKD stage. Living kidney donors have renal function consistent with early stage CKD and concern has been raised about their cardiovascular risk. No studies to date have investigated the presence of coronary microvascular dysfunction in living kidney donors.

**Methods:**

25 healthy controls and 23 living kidney donors were recruited and underwent assessment with transthoracic echocardiography, Doppler CFVR, myocardial contrast echocardiography and serum multiplex immunoassay panels.

**Results:**

Doppler CFVR was significantly reduced in living kidney donors compared to controls (mean CFVR 3.4 ± 0.7 vs 3.8 ± 0.6, mean difference 0.4 95% confidence interval 0.03–0.8, *p* =.036). Quantitative myocardial contrast echocardiography showed a trend towards reduced coronary flow reserve in living kidney donors. Compared to controls, living kidney donors had higher serum high sensitivity C reactive peptide (hsCRP) and lower levels of uromodulin.

**Conclusions:**

This is the first study of CFVR in living kidney donors. We have shown that the modest drop in estimated glomerular filtration rate in living kidney donors is associated with lower values of Doppler CFVR compared to controls, suggesting that isolated reductions in renal function may lead to altered microvascular function. The increase in hsCRP and reduction in uromodulin suggests that chronic subclinical inflammation may contribute to altered microvascular function in this population.

## Highlights

• Coronary flow velocity reserve is reduced in living kidney donors compared to controls.
• Markers of inflammation are elevated in living kidney donors.
• Inflammation may cause coronary microvascular dysfunction in living kidney donors.
• Living kidney donors need aggressive cardiovascular risk factor management.

## 1 Introduction

Kidney transplantation is the most effective form of renal replacement therapy and is associated with significant health benefits for the recipient, including improved blood pressure (BP) control, and reduced all-cause and cardiovascular mortality [1]. Given the shortage of cadaveric donors, there is a worldwide drive to increase rates of living kidney donation, which now accounts for approximately 30% of transplants in the United Kingdom (UK) [2]. Living kidney donors (LKD) provide a unique model of reduced estimated glomerular filtration rate (eGFR) without progressive kidney disease or confounding comorbidities. After unilateral nephrectomy, most donors will have an eGFR consistent with stage 2–3 chronic kidney disease (CKD) [3]. Although long term evidence shows that living kidney donation is safe, the possible cardiovascular risks of living kidney donation remain unclear. Previous studies of LKD have shown small but significant changes in cardiovascular structure and function at 1 year after donation [4,5]. Although the majority of studies, including a recent meta-analysis, have not shown any increased mortality compared to the general population [6,7], Mjoen et al raised concerns about long term mortality in LKD when compared to a highly selected control group who met the eligibility criteria for living kidney donation [8].

There is growing interest in the role that coronary microvascular dysfunction (CMD) may play in the increased cardiovascular risk seen in CKD [9]. Coronary flow reserve (CFR) is a widely reported parameter of microvascular function and is primarily a measure of the ability of the microcirculation to respond to vasodilatory stimuli. In normal subjects, coronary flow should at least double with hyperaemia, so a CFR <2 is considered abnormal [9]. Multiple studies have shown a graded inverse relationship between CFR and CKD stage, and this has prognostic significance [[9], [10], [11], [12], [13], [14]]. Both CFR and its surrogate marker CFVR (coronary flow velocity reserve) can be reliably measured using non-invasive contrast enhanced echocardiography techniques [[15], [16], [17]].

Reduced CFR is seen even in early CKD (stages 1–3), a level of eGFR often present in LKD [[10], [11], [12]]. Given the increasing numbers of LKD worldwide, it is important to assess whether unilateral nephrectomy is associated with impaired microvascular function, which may have long term implications for cardiovascular risk in donors. The Chronic Renal Impairment in Birmingham Coronary Flow Reserve (CRIB-FLOW) study was designed to assess coronary microvascular function in LKD and to look for associations between CFVR and markers of inflammation and fibrosis.

## 2 Methods

### 2.1 Study population

Between May 2019 and February 2020, 23 LKD and 25 healthy controls were enrolled in the CRIB-FLOW study at the Queen Elizabeth Hospital, Birmingham (QEHB) – Supplementary Fig. 1. Participants were > 18 years of age and provided written informed consent. The study was carried out in accordance with the principles of the Declaration of Helsinki. Donors were recruited from the LKD registry at QEHB. Healthy controls, of a similar age and gender, were recruited from staff members and control subjects from the Chronic Renal Impairment in Birmingham – Donor (CRIB-Donor) study [4].

Kidney donors were >12 months post-donation. Healthy controls had eGFR >90ml/min/1.73m^2^ or eGFR 60-90ml/min/1.73m^2^ and no significant proteinuria or signs of kidney damage. The Chronic Kidney Disease Epidemiology Collaboration formula was used to calculate eGFR [18]. Exclusion criteria were: pregnancy, diabetes mellitus, uncontrolled hypertension, ischaemic heart disease, moderate/severe valvular heart disease and contraindication to adenosine or sulfur hexafluoride contrast agent (SonoVue, Bracco, Milan, Italy). The study was reviewed and approved by the West Midlands – Solihull Research Ethics Committee (19/WM/0066) and registered with ClinicalTrials.gov (NCT04014127).

### 2.2 Blood pressure

Supine and sitting office BP were measured using an automatic BP monitor. The average of five readings taken over five minutes was used.

### 2.3 Transthoracic echocardiography (TTE)

Two-dimensional echocardiography was performed by a single experienced cardiologist (AR) using a Philips iE33 machine (Philips, Eindhoven, Netherlands) with S5–1 transducer for TTE and myocardial contrast echocardiogram (MCE) studies and S8–3 transducer for CFVR measurements.

Left ventricular mass was estimated using the Cube formula and indexed for body surface area [19]. Left ventricular volumes and ejection fraction were measured using the Simpson's biplane method [19]. Diastolic function was quantified using multiple parameters [20]. Global longitudinal strain was assessed in the 3 standard apical views using speckle tracking.

### 2.4 Doppler coronary flow velocity reserve

**Supplementary Fig. 2:**
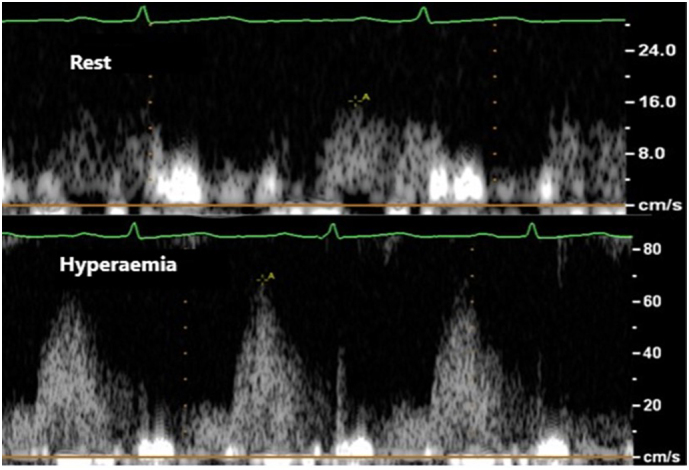
Rest and hyperaemic coronary flow velocity in the distal left anterior descending artery.

Subjects were asked to abstain from caffeine for 24 h prior to the study. The left anterior descending artery (LAD) was identified on colour Doppler in the anterior inter-ventricular sulcus using a modified apical 2-chamber view (distal LAD) or a low parasternal short axis view (mid LAD) as previously described [15]. Pulse wave Doppler signals of LAD flow were recorded at rest and at hyperaemia, maintaining an identical probe position and angle. SonoVue was used, if needed, to identify LAD flow and accentuate Doppler signals. Adenosine was infused, with BP and electrocardiogram (ECG) monitoring, at a rate of 140micrograms/kg/min for 3 min to induce hyperaemia. Peak diastolic coronary flow velocity (CFV) was calculated at rest and hyperaemia – Supplementary Fig. 2. CFVR was calculated as hyperaemic CFV/rest CFV. For each variable in the CFVR calculation, the highest values of 3 cardiac cycles were averaged.

### 2.5 Myocardial contrast echocardiography

Myocardial contrast echocardiography was performed as previously described [17]. Briefly, images were taken in the 3 standard apical views using low-power continuous MCE at a mechanical index (MI) of 0.1. SonoVue was infused at a rate of 70-100 ml/h using an infusion pump that oscillates gently throughout the infusion to ensure that microbubbles remain in suspension (Vueject, Bracco, Milan, Italy). The infusion rate was adjusted to ensure adequate myocardial opacification without attenuation. The focus was set at the level of the mitral valve but moved towards the apex to avoid near-field artefact. Triggered high MI (1.0) flash echocardiography at end-systole was performed to destroy microbubbles in the myocardium and to observe replenishment. End-systolic frames of up to 10 cardiac cycles were captured in each view. Rest and adenosine vasodilator stress images were recorded. Stress images were reviewed for any regional wall motion abnormalities or any sub-endocardial perfusion defects suggesting myocardial ischaemia.

### 2.6 Quantitative myocardial contrast echocardiography

The QLab system (Philips, Eindhoven, Netherlands) was used to quantify MCE. The left ventricle was segmented using a 16-segment model [19]. Regions of interest were placed across the entire thickness of the myocardium in the 10 mid and apical segments, taking care to exclude the high-intensity endocardial and epicardial borders. Basal segments were excluded due to high rates of artefact. Segments were also excluded if there was artefact, inadequate microbubble destruction, attenuation, or a wide variation in contrast intensity. A minimum of 6 quantifiable segments was necessary for the study to be included in analysis.

The QLab software automatically generated background-subtracted plots of contrast intensity vs time which were fitted to an exponential function y = A(1 − e − β^t^). From this, peak myocardial contrast intensity (A - representing myocardial blood volume) and the slope of the replenishment curve (β - depicting mean microbubble velocity) could be derived. The product of Axβ equals myocardial blood flow (MBF). LAD MBF (average of mid anteroseptal, apical septal, mid anterior and apical anterior segments) and global MBF (average of all ten segments) were calculated at rest and at stress. CFR was calculated as MBF_stress_/MBF_rest_ [17].

### 2.7 Blinded analysis

Echocardiograms were stored under an anonymous code and analysed offline using commercially available software (IntelliSpace Cardiovascular, Philips, Eindhoven, Netherlands). The TTE, CFVR and MCE studies were all analysed by a single investigator (AR) blinded to study group. Ten randomly selected studies had repeat blinded Doppler CFVR analysis by the same investigator to assess intra-observer variability.

### 2.8 Serum biomarkers

Serum biomarkers of inflammation, myocardial stretch, cardiac fibrosis and markers associated with left ventricular hypertrophy (LVH) were tested in both LKD and controls. N-terminal pro brain natriuretic peptide was assayed using the Alere point of care assay (Alere, Massachusetts, USA). High sensitivity C-reactive peptide (hsCRP) was assayed using the Architect MULTIGENT CRP Vario assay (Abbott, Illinois, USA). The fluorescence responses of 16-analytes were obtained using Human Magnetic Luminex® Asssays (R&D Systems, Minneapolis, MN, USA) and the Bio-RAD Bio-Plex™ 200 system for analysis. Concentrations were calculated using the Bio-Plex Software Manager™ (version 6.1) generated standard curves and a 5PL logistic curve fitting technique as per the manufacturer's instructions [21].

### 2.9 Endpoints & sample size justification

The primary endpoint was difference in mean Doppler CFVR between controls and LKD. Based on previous data by Imamura et al [10] [CFVR for controls (3.8 ± 0.4), CFVR for CKD stage 2 (3.2 ± 0.7), CFVR for CKD stage 3 (3.0 ± 0.6)] - we estimated that 22 patients in each group would provide 80% power with an alpha value of 0.05 to demonstrate a difference in Doppler CFVR of 0.6 between controls and LKD. Difference in CFR by MCE was the secondary endpoint.

### 2.10 Statistical analysis

Statistical analysis was carried out using SPSS version 26 (SPSS Inc., Chicago, Illinois). Data normality was assessed using the Shapiro-Wilk test. Continuous variables are expressed as mean ± standard deviation (SD) for parametric data or median (interquartile range) for non-parametric data. Unpaired group comparisons for continuous data were made using the unpaired *t*-test or the Mann-Whitney *U* test. Unpaired categorical data were compared using Fisher's exact test. Correlation was assessed using the Pearson correlation coefficient. Statistical tests were 2-tailed, and a *p* value <.05 was considered statistically significant.

## 3 Results

### 3.1 Subject characteristics

Baseline demographic, laboratory and haemodynamic data are presented in Table 1. Median time from donation in LKD was 30 months (interquartile range 24–67 months). There were no significant differences in demographic variables between controls and LKD. One LKD was on anti-hypertensive therapy. Two controls and 1 LKD were on statin therapy. Of the remaining 18 participants with total cholesterol >5mmol/L, only 1 LKD and 2 controls met UK criteria for primary prevention statin therapy (QRISK3 10 year risk >10%) [22].

**Table 1:** Demographic, laboratory and haemodynamic variables.

	Controls (*n* = 25)	Donors (*n* = 23)	*p* value
*Demographics*			
Age (years)	41 ± 10	46 ± 10	0.098
Male *n*(%)	18 (72)	16 (70)	0.853
Caucasian *n*(%)	15 (60)	18 (78)	0.173
BMI (kg/m^2^)	25.6 ± 2.3	26.8 ± 4.2	0.230
Smoker n(%) – Current	2 (8)	3 (13)	0.905
Ex	5 (20)	4 (17)	
Never	18 (72)	16 (70)	
Hypertension *n*(%)	1 (4)	1 (4)	1.0
Hypercholesterolaemia *n*(%)	8 (32)	13 (57)	0.145
ACE inhibitors *n*(%)	0 (0)	1 (4)	0.479
Statin therapy *n*(%)	2 (8)	1 (4)	1.0
Time from donation (months)	n/a	30 (24–67)	n/a
*Laboratory data*			
Haemoglobin (g/l)	146 ± 11	141 ± 10	0.198
Urea (mmol/l)	5.0 ± 1.3	5.7 ± 1.1	0.061
Creatinine (μmol/l)	80 ± 17	107 ± 15	**<0.001**
eGFR (ml/min/1.73m^2^)	99 (91–112)	68 (64–72)	**<0.001**
ACR (mg/mmol)	0.9 (0–2.1)	0.9 (0–1.8)	0.298
Phosphate (mmol/l)	1.13 ± 0.17	1.03 ± 0.17	**0.042**
Corrected calcium (mmol/l)	2.33 ± 0.08	2.36 ± 0.08	0.152
PTH (μmol/l)	5.7 ± 2.1	6.6 ± 2.0	0.237
Total cholesterol (mmol/l)	4.6 (4.0–5.2)	5.1 (4.8–5.6)	0.06
LDL cholesterol (mmol/l)	2.7 ± 1.0	3.2 ± 0.8	0.06
NT-proBNP (ng/l)	40 (22–69)	54 (24–95)	0.391
Detectable CRP *n*(%)	7 (29)	18 (73)	**0.01**
hsCRP (mg/l)	0.63 (0.41–0.86)	1.31 (0.92–2.0)	**0.006**
Urate (μmol/l)	332 ± 84	366 ± 82	0.158
Renin (mIU/l)	21.2 (16.9–35.6)	17.9 (13.4–35.5)	0.324
Aldosterone (μmol/l)	161 (129–225)	129 (44–222)	0.156
*Haemodynamic data*			
Systolic BP (mmHg)	116 ± 11	115 ± 12	0.835
Diastolic BP (mmHg)	76 ± 10	76 ± 10	0.816
Heart rate (bpm)	71 ± 12	65 ± 11	0.066

Data are presented as mean ± SD or median (IQR). BMI – body mass index, ACE – angiotensin converting enzyme, eGFR – estimated glomerular filtration rate, ACR – albumin creatinine ratio, PTH – parathyroid hormone, LDL – low density lipoprotein, NT-proBNP – n terminal pro brain natriuretic peptide, CRP – C reactive peptide, hsCRP – high sensitivity C reactive peptide, BP – blood pressure.

There was a significant difference in creatinine and eGFR between controls and donors. 3/23 (13%) donors had eGFR consistent with stage 3 CKD while the remainder had eGFR in the range of CKD stage 2. Serum phosphate was significantly lower in LKD. Detectable C reactive peptide (CRP) and median high sensitivity C reactive peptide (hsCRP) were both significantly higher in LKD.

There were no significant differences in TTE parameters between controls and LKD - Table 2. One individual had previously undiagnosed severe aortic regurgitation detected on baseline TTE. Markers of systolic and diastolic function were similar between the two groups.

**Table 2:** Echocardiographic parameters.

	Controls (*n* = 25)	Donors (*n* = 23)	*p* value
IVSD (mm)	10 (9–11)	10 (8–11)	0.106
LVIDD (mm)	44 ± 4	44 ± 5	0.946
PWD (mm)	9 (8–10)	9 (8–10)	0.732
LVIDS (mm)	28 ± 3	29 ± 4	0.470
Fractional Shortening (%)	36 (31–38)	32 (31–36)	0.201
LVEDVi (ml/m^2^)	46 ± 8	47 ± 10	0.716
LVESVi (ml/m^2^)	17 (14–19)	18 (13−22)	0.713
EF (%)	62 (60–65)	61 (57–65)	0.305
TAPSE (mm)	21 ± 3	20 ± 3	0.168
GLS (%)	−19 ± 3	−19 ± 3	0.849
LV mass index (g/m^2^)	71 (62–88)	69 (57–76)	0.307
LV geometry n(%) – normal geometry	17 (68)	14 (61)	0.439
Concentric remodelling	6 (24)	9 (39)	
Eccentric hypertrophy	1 (4)		
Concentric hypertrophy	1 (4)		
Left atrial volume index (ml/m^2^)	19.3 ± 4.3	20.5 ± 6.8	0.477
E/A ratio	1.2 ± 0.3	1.1 ± 0.2	0.184
E/e’	6 (5–8)	6 (6–7)	0.655

Data are presented as mean ± SD or median (IQR). IVSD – interventricular septal diameter, LVIDD – left ventricular internal diameter diastole, PWD – posterior wall diameter, LVIDS – left ventricular internal diameter systole, LVEDVi – indexed left ventricular end diastolic volume, LVESVi – indexed left ventricular end systolic volume, EF – ejection fraction, TAPSE – tricuspid annular plane systolic excursion, GLS – global longitudinal strain, LV – left ventricular.

### 3.2 Doppler coronary flow velocity reserve

Doppler CFVR was not attempted in the subject with severe aortic regurgitation on baseline TTE. The technique was feasible in 46/47 (99%) of subjects in which it was attempted. One subject did not tolerate adenosine and thus no hyperaemic measurements were available. One subject was subsequently excluded from CFVR analysis due to the new finding of thyrotoxicosis on serum biochemistry. Final Doppler TTE CFVR data were available in 22 controls and 23 LKD. SonoVue was used in 31/45 (69%) cases. There was no significant intra-observer variability for offline Doppler CFVR analysis (ICC 0.99 95% confidence interval 0.956–0.998, *p* < .001).

**Fig. 1:**
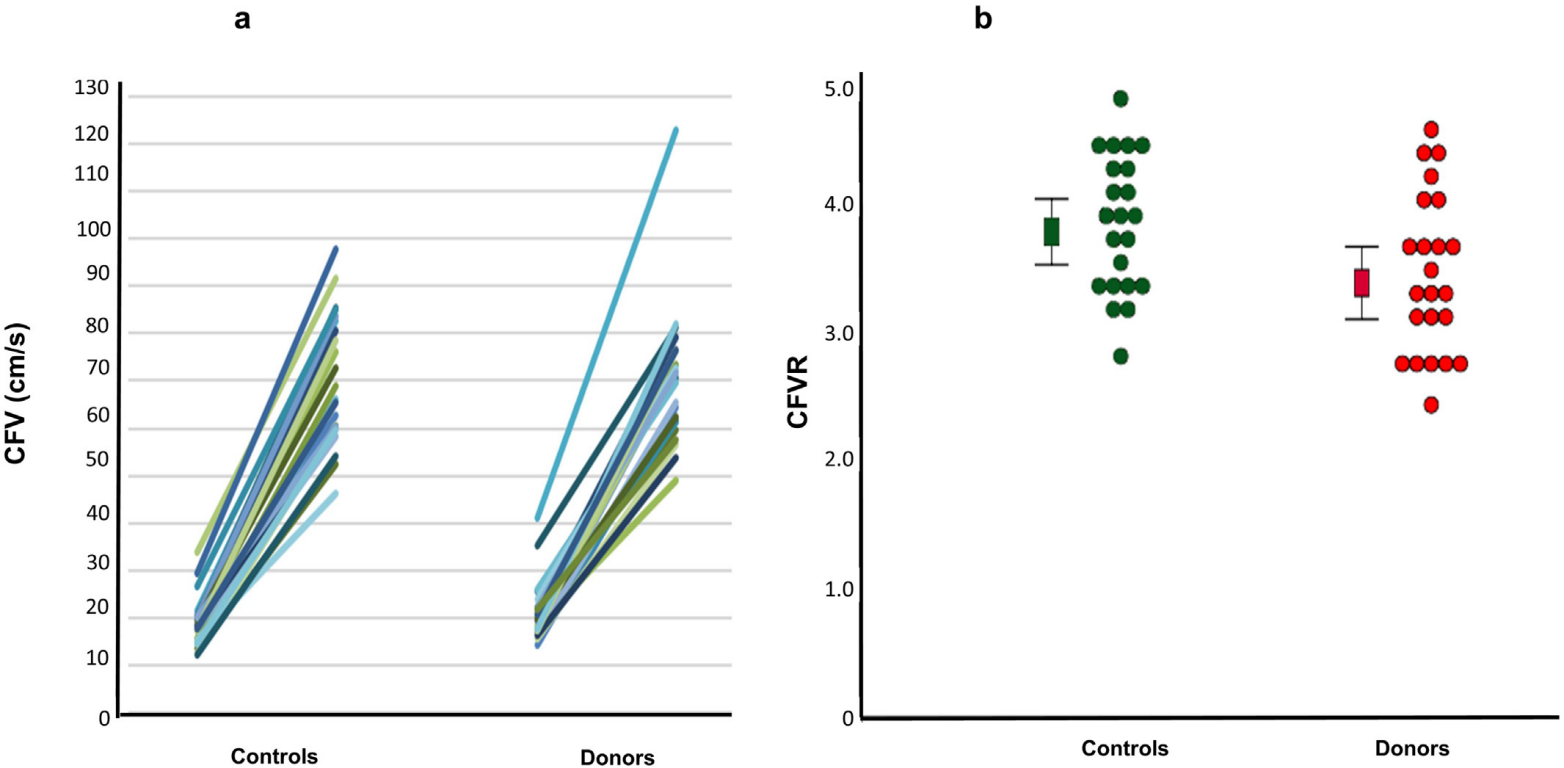
1a – Coronary flow velocity at rest and at hyperaemia in controls and living kidney donors. 1b – Doppler coronary flow velocity reserve in controls and living kidney donors. Squares represent mean. Error bars represent 95% confidence intervals. Circles represent individual CFVR measurements. CFV – coronary flow velocity, CFVR – coronary flow velocity reserve.

Resting CFV in donors was slightly higher than in controls, although this was not statistically significant [median CFV 19.9 (17.4–22.2) vs 18.1 (15.6–20.4), *p* = .114]. Hyperaemic CFV did not differ (mean CFV 70.2 ± 14.6 vs 70.5 ± 13.8, *p* = .944) – Fig. 1a. CFVR was significantly reduced in LKD compared to controls (mean CFVR 3.4 ± 0.7 vs 3.8 ± 0.6, mean difference 0.4 95% confidence interval 0.03–0.8, *p* = .036) – Fig. 1b. Although no subjects in our study had CFVR<2, 6/23 (26%) LKD had CFVR ≤2.7 (the lowest CFVR value in controls). There was a modest significant correlation between eGFR and CFVR (*r* = 0.3 *p* = .034).

### 3.3 Myocardial contrast echo

No subjects had stress induced wall motion abnormalities or perfusion defects on qualitative MCE. Quantitative MCE was possible in only 14 controls and 19 LKD. Both LAD CFR and global CFR were numerically lower in LKD, although this was not statistically significant – LAD CFR [median CFR 3.4 (2.6–5.0) vs 2.7 (2.2–3.9), *p* = .212] and global CFR [median CFR 3.4 (2.2–3.8) vs 3.0 (2.3–4.2), *p* = 1.0].

### 3.4 Multiplex immunoassay

The results of the Multiplex immunoassay are shown in Table 3. One control did not provide blood for immunoassay analysis. There were no significant differences between controls and LKD in the assays tested, apart from uromodulin which was significantly lower in LKD.

**Table 3:** Results of human magnetic luminex assay.

Assay	*Controls (n* *=* *24)*	*Donors (n* *=* *23)*	*p value*
Angiopoetin-2 (pg/ml)	1518 (1260–2006)	1348 (1143–1865)	0.322
Atrial natriuretic peptide (pg/ml)	4730 (3449–6145)	5778 (3653–8248)	0.268
Detectable IL-10 *n*(%)	11 (44)	11 (48)	0.790
Detectable KIM-1 *n*(%)	9 (36)	11 (48)	0.406
Galectin-3 (ng/ml)	0.9 (0.8–1.2)	1.1 (0.8–1.3)	0.317
IL-1ra (pg/ml)	522 (356–655)	503 (340–703)	0.807
IL-6 (pg/ml)	1.26 (0.82–1.86)	1.26 (0.97–1.81)	0.661
IL-8 (pg/ml)	12.3 (8.4–25.5)	11.3 (8–29.1)	0.992
Leptin (ng/ml)	5.7 (3.0–11.1)	4.9 (3.2–8.5)	0.865
MCP-1 (pg/ml)	378 (298–537)	391 (325–480)	0.670
MMP-9 (pg/ml)	9118 (6465–13,292)	9928 (7374–19,628)	0.360
NGAL (ng/ml)	15.5 (14.0–16.6)	16.7 (14.4–18.3)	0.187
ST2 (ng/ml)	12 (9–16)	10 (6–18)	0.444
TNFα (pg/ml)	3.5 (2.53–4.22)	3.37 (2.59–4.28)	0.924
Uromodulin (ng/ml)	98 ± 43	67 ± 35	**0.009**
VEGF (pg/ml)	48 (24–60)	65 (41–93)	0.101

Data are presented as mean ± SD or median (IQR). IL-10 – interleukin-10, KIM-1 – kidney injury molecule 1, IL-1ra – interleukin 1 receptor antagonist, IL-6 – interleukin-6, IL-8 – interleukin-8, MCP-1 – monocyte chemoattractant protein, MMP-9 – matrix metallopeptidase 9, NGAL – neutrophil gelatinase associated lipocalin, TNFα – tumour necrosis factor alpha, VEGF – vascular endothelial growth factor.

## 4 Discussion

This is the first study of CFVR in LKD. Despite only modest reductions in eGFR, LKD had a significantly lower Doppler CFVR than controls. These results suggest that reductions in renal function alone can lead to altered microvascular function. Reassuringly, no subjects in our cohort had CFVR<2, which is known to be a poor prognostic marker [13].

Previous studies using Doppler TTE have shown intra-subject variations in CFVR of 0.3–0.45 [15,23]. Given that the difference in CFVR between controls and LKD in our study was similar to this value, we cannot fully exclude the possibility that this difference was due to chance. However, our sample size was adequate and we would expect similar variability of CFVR measurements in both groups. Furthermore, the magnitude of difference between our controls and LKD is similar to the previously demonstrated difference between controls and subjects with CKD stage 2 – a group that have similar renal function to LKD [10].

The wider variances in CFR by MCE among our subjects suggest that our study may have been underpowered for this secondary endpoint. Adenosine can cause uncomfortable dyspnoea and chest wall movement that compromises the image quality needed for optimal MCE quantification. Previous studies have used intravenous dipyridamole [17], which has fewer respiratory side effects, but was not available in our hospital. Coronary flow reserve by MCE was measurable in 69% of our cohort, which is consistent with previous studies showing that quantitative MCE using adenosine is feasible in only 33–75% of patients [24,25]. Despite these limitations, our MCE data showed a trend towards reduced CFR in LKD, which is consistent with our Doppler CFVR data. We chose Doppler CFVR as our primary endpoint as the technique is feasible and highly reproducible even with limited image quality [15].

The mechanisms of microvascular dysfunction in LKD are not clear but abnormalities of both structure and function may be present. Animal models have demonstrated reduced capillary length and density in the hearts of rats who underwent subtotal nephrectomy and evidence of fibrosis and diastolic dysfunction in rats after uni-nephrectomy [26,27].

The reduced CFVR among LKD in our study was predominantly due to a higher baseline CFV in LKD, with similar maximal hyperaemic values. Elevated resting CFV is seen in CKD and hypertension and has been attributed to increased oxygen demand as a result of hypertension, LVH and diastolic dysfunction [10,28]. Elevated resting CFV may also be related to increased sympathetic nervous system (SNS) activity which causes vasoconstriction of vascular smooth muscle cells, leading to increased coronary vascular resistance and a decrease in coronary perfusion pressure [29]. Increased SNS activity is seen in early CKD but has not been studied in LKD [30]. In addition, the reduced CFVR in LKD also reflects a diminished hyperaemic response to adenosine, indicating impaired vasodilatation in the coronary microcirculation, where adenosine predominantly has its effect [31]. Adenosine-induced vasodilatation is at least partially mediated by nitric oxide release from the endothelium [32], suggesting that endothelial dysfunction may be a contributory mechanism for CMD in LKD. Studies in early CKD have shown that endothelial dysfunction is common and is associated with poor prognosis [33,34]. To date, there are no studies of endothelial function in LKD but the CENS study, which is currently recruiting, will provide a comprehensive assessment of endothelial function in LKD [35].

Chronic inflammation in systemic inflammatory conditions is associated with CMD [36]. Both detectable CRP and mean hsCRP were significantly higher in LKD. An inflammatory response has been shown in the early post-operative period in LKD with an 80-fold increase in CRP in the first week after nephrectomy [37]. Longer term data on chronic inflammation in LKD are conflicting. Huan et al showed no increase in inflammatory markers in LKD at 6 months post donation [38]. However, Moody et al showed an increase in the prevalence of detectable CRP in LKD at 12 months post donation [4]. The elevated hsCRP suggests that a pattern of subclinical chronic inflammation may be present in LKD, as it is in subjects with CKD [39]. Uromodulin, a glycoprotein secreted by the thick ascending limb of the loop of Henle, may play a role in this process. In a normally functioning kidney, uromodulin may have a protective anti-inflammatory role through neutralisation of urinary cytokines. As renal function declines, so does uromodulin. In the presence of tubular damage, as seen in CKD, the reduction in uromodulin may have a pro-inflammatory effect by activating NLRP3 dependent IL-1β secretion and subsequent induction of other pro-inflammatory cytokines [40]. It is possible that the raised CRP, hsCRP and uromodulin in LKD were chance findings due to the large number of variables tested. After adjustment with a Bonferroni correction for multiple endpoints, they fail to reach statistical significance. However, this correction has been subject to criticism [41], and as CKD is characterised by systemic inflammation, there are plausible reasons why subjects with reduced kidney function due to uni-nephrectomy might also exhibit a pro-inflammatory state. The role of inflammation after nephrectomy warrants further research.

The clinical significance of our findings needs further investigation. It is possible that this small reduction in coronary microvascular function in LKD may not have clinical sequelae and is an epi-phenomenon related to persistent low-grade inflammation after uni-nephrectomy. However, there is increasing evidence of a possible role for CMD in the development of heart failure with preserved ejection fraction and uraemic cardiomyopathy [9]. In CKD, the presence of CMD is associated with abnormalities of diastolic function and indices of systolic deformation, as well as adverse cardiovascular outcomes including death, myocardial infarction and heart failure hospitalisation [14]. Thus, a paradigm has been suggested in which risk factors such as inflammation and hypertension lead to CMD, which in turn causes diffuse ischaemia and adverse left ventricular re-modelling, leading eventually to uraemic cardiomyopathy with its adverse prognosis [9]. Our results should stimulate long term studies of LKD to determine their subsequent risk of the development of diastolic dysfunction, adverse left ventricular remodelling and uraemic cardiomyopathy. As long-term cardiovascular risk in LKD remains unclear and CMD carries a poor prognosis, baseline assessment of coronary microvascular function may be worthwhile in potential kidney donors, to help identify individuals who are at increased cardiac risk from kidney donation.

## 5 Limitations

Similar to other non-invasive studies of CFVR, we could not fully exclude coronary artery disease (CAD) in our cohort without coronary angiography (either computed tomography or invasive). However, all subjects had normal ECG and no coronary distribution perfusion defect or regional wall motion abnormality on vasodilator MCE – a highly sensitive and specific technique for the diagnosis of flow limiting CAD [42]. Thus we have strong evidence that there was no myocardial ischemia due to CAD in our cohort.

Our cohort was predominantly male and Caucasian, limiting the generalisability of our findings to the wider LKD population. However, UK data does show that the majority of LKD are Caucasian [2], and it has previously been shown that there are similar rates of CMD among men and women [43].

Finally, our study was cross-sectional in design, meaning that causation cannot be definitively demonstrated. Future longitudinal work examining CFVR pre- and post-nephrectomy is needed to confirm the observation seen in our study.

## 6 Conclusions

Our study has shown that Doppler CFVR is reduced in LKD compared to healthy controls, suggesting subclinical impairment of microvascular function. Although current data suggests that living kidney donation remains extremely safe, our study highlights the importance of long-term follow-up and aggressive risk factor management to detect subtle cardiovascular changes and to minimise any future cardiovascular morbidity and mortality in this population. The role of chronic inflammation in LKD also needs further examination.

**The following are the supplementary data related to this article.Supplementary Fig. 1:**
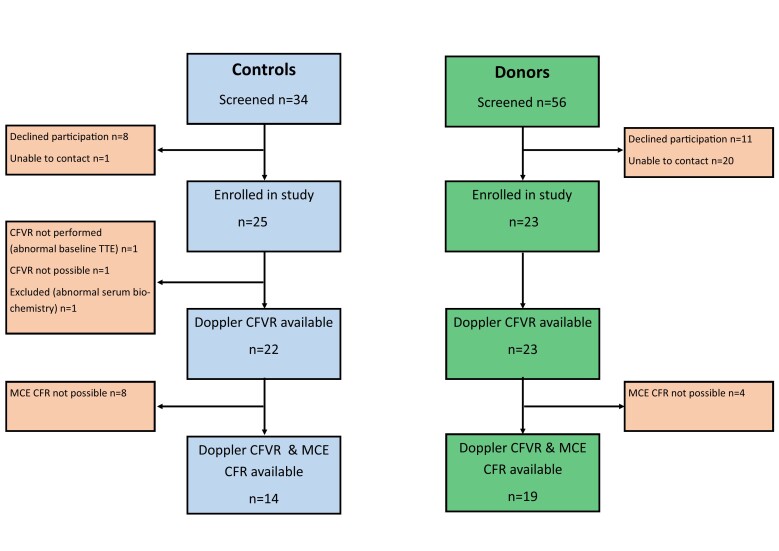
CONSORT statement for CRIB FLOW study. TTE – transthoracic echocardiogram, CFVR – coronary flow velocity reserve, CFR – coronary flow reserve, MCE – myocardial contrast echocardiogram.

## Funding

The CRIB-FLOW study was funded by research grants from University Hospitals Birmingham Charity and the Metchley Park Medical Society. AMP, LCP and JPL are holders of British Heart Foundation Clinical Research Training Fellowships (FS/16/73/32314, FS/18/29/33554 and FS/19/16/34169 respectively). LF has received support via the Institute of Cardiovascular Sciences, University of Birmingham: Fondation Leducq & British Heart Foundation Accelerator Award (AA/18/2/34218).

## Author statement

All authors take responsibility for all aspects of the reliability and freedom from bias of the data presented and their discussed interpretation.

## Declaration of Competing Interest

LF has received institutional research grants and non-financial support from European Union, British Heart Foundation, Medical Research Council (UK), DFG and Gilead. LF is listed as inventor on two patents held by University of Birmingham (Atrial Fibrillation Therapy WO 2015140571, Markers for Atrial Fibrillation WO 2016012783). No other authors have any conflict of interest to declare.
